# Functional Neural Underpinnings of Increased Falls Risk

**DOI:** 10.1093/geroni/igaa057.1581

**Published:** 2020-12-16

**Authors:** Meishan Ai, Chun-Liang Hsu, Teresa Liu-Ambrose

**Affiliations:** 1 Northeastern University, Boston, Massachusetts, United States; 2 Harvard Medical School, Roslindale, United States; 3 University of British Columbia, Vancouver, British Columbia, Canada

## Abstract

Impaired cognition, especially in the domain of executive functions, is a major risk factor for falls in older adults. However, the underlying neural mechanism of the association between cognitive function and falls risk remains unclear. The current study compared the performance on mobility and cognitive assessments, and brain activation under Stroop task between fallers (>=2 falls in the past year) and non-fallers (0 or 1 fall in the past year). We found that during the incongruent condition of Stroop task, higher activation in precuneous, frontal and temporal areas was observed in fallers, while they showed comparable task performance as non-fallers. In addition, the contrast between congruent and incongruent conditions showed fallers exhibited increased activation in middle frontal region (z>1.7, P<0.05). Further, through mediation analysis, our data revealed that brain activation in temporal and frontal-paracingulate regions mediated the relationship between Montreal Cognitive Assessment and number of falls (confidence interval = 95%), after controlling for age and sex. Overall, our findings suggested that lower neural efficiency may be an underlying mechanism by which cognitive function is associated with falls risk.

